# Preventive action of ketamine alone and in combination with antifungals on *Candida* biofilm formation in catheters

**DOI:** 10.1007/s10123-026-00851-y

**Published:** 2026-07-01

**Authors:** Cecília Rocha da Silva, Lívia Gurgel do Amaral Valente Sá, Lisandra Juvêncio da Silva, Maria Safira Mendes Martins, Maria Janielly Castelo Branco Silveira, Érica Rayanne Mota da Costa, Vitória Pessoa de Farias Cabral, Daniel Sampaio Rodrigues, Lara Elloyse Almeida Moreira, Vitória Monteiro Matos, Vitória Girão dos Santos, Hélio Vitoriano Nobre, João Batista de Andrade Neto

**Affiliations:** 1 School of Pharmacy, Laboratory of Bioprospection in Antimicrobial Molecules (LABIMAN), Federal University of Ceará, Fortaleza, CE Brazil; 2 Drug Research and Development Center (NPDM), Federal University of Ceará, Fortaleza, CE Brazil; 3 Christus University Center (UNICHRISTUS), Fortaleza, CE Brazil

**Keywords:** Biofilm, *Candida*, Catheter, Ketamine

## Abstract

**Purpose:**

This study investigates the repurposing potential of the anesthetic ketamine (KET) as a preventive agent, both alone and in combination with the azoles fluconazole (FLC) and itraconazole (ITR), against *Candida* spp. biofilms adhering to fragments of peripheral venous catheters.

**Methods:**

The activity was assessed in vitro through cell viability and colonization assays of impregnated catheter segments, while morphological alterations were analyzed using scanning electron microscopy (SEM).

**Results:**

KET, both alone and in association with ITR, significantly reduced (*p* < 0.05) the adherence of *C. albicans* to the impregnated fragments. Furthermore, the combinations KET + FLC and KET + ITR frequently exhibited greater efficacy in reducing biofilm viability than the agents used individually, suggesting additive/synergistic interactions. SEM revealed structural damage to the treated fungal cells.

**Conclusion:**

These findings indicate that KET exhibits preventive activity against *Candida* spp. biofilms, including on catheter surfaces, and may enhance the activity of azoles, positioning it as a promising candidate for repurposing in the treatment of biofilm-associated fungal infections.

## Introduction

Bloodstream infections caused by *Candida* species, known as candidemia, are a significant global health threat, associated with high morbidity and mortality rates, particularly in hospital settings (Pappas et al. 2015, 2018; Agnelli et al. 2022). The genus *Candida* is among the leading causes of nosocomial sepsis worldwide (Pappas et al. 2018). Although *Candida albicans* remains the most frequently isolated fungal pathogen in humans, the incidence of infections caused by non-*albicans Candida* (NAC) species, such as *Nakaseomyces glabrata*, *Candida parapsilosis*, *Candida tropicalis*, and *Candida krusei*, has increased considerably in recent decades, often exhibiting intrinsic or acquired antifungal resistance profiles (Whibley and Gaffen 2015; Gómez-Gaviria et al. 2023).

The virulence of *C. albicans*, a common commensal species of the human microbiota, is intrinsically linked to its ability to undergo morphological transition into filamentous forms (hyphae), and especially to form biofilms (Gulati and Nobile 2016; Lopes and Lionakis 2022). Biofilms are structured microbial communities embedded in a matrix of extracellular polymeric substances (EPS), which provide protection against host defenses and drastically increase their tolerance to antifungal drugs (Gulati and Nobile 2016; Karygianni et al. 2020).

These structures are frequently associated with invasive medical devices, such as intravenous catheters, which serve as niches for colonization and entry into the bloodstream, representing a major risk factor for candidemia in hospitalized and immunocompromised patients or those undergoing prolonged antibiotic therapy (Alves et al. 2020; D’Enfert et al. 2021; Rinawati et al. 2022).

The clinical and economic burden of invasive fungal infections (IFIs), including candidemia, is substantial. In Brazil, for instance, studies indicate that the incidence may reach up to 5.4 cases per 10,000 patient-days, with associated costs approaching USD 150,000 per episode because of the need for extended hospital stays and costly antifungal therapies. Globally, IFIs are estimated to cause approximately 1.5 million deaths annually, underscoring the urgency for more effective therapeutic strategies (Pfaller and Castanheira 2016; de Oliveira et al. 2021; Puumala et al. 2024).

A major challenge in the management of fungal infections lies in the limited therapeutic arsenal currently available, which is restricted to a few major classes such as azoles, polyenes, and echinocandins (Fisher et al. 2022; Vanreppelen et al. 2023). This limitation, in stark contrast to the vast range of antibacterial options, not only complicates treatment but also contributes to the growing threat of antifungal resistance (Vanreppelen et al. 2023).

Although the development of new antifungal agents is a rational response to the scarcity of effective therapeutic classes, it is a slow, complex and costly process. The approval of a new anti-infective agent may take five to seven years following the initiation of clinical trials, with outlays frequently exceeding hundreds of millions of dollars. Thus, it is important to direct efforts toward the development of novel therapeutic alternatives and the prevention of fungal infections, particularly those caused by resistant strains of *Candida* spp. (Pereira et al. 2021; Fisher et al. 2022).

In this context, drug repurposing, which is the identification of new therapeutic applications for medications already approved for other indications, emerges as a fast and cost-effective strategy to expand treatment options (Ahmad et al. 2024). This approach is being explored as a therapeutic alternative in challenging areas like the treatment of fungal biofilms (de Barros et al. 2020).

Furthermore, drug repurposing has proven to be effective in the prevention of infections associated with medical devices such as catheters. The incorporation of these medications reduces surface colonization of such devices, thereby preventing biofilm formation (Sidrim et al. 2019; Silva et al. 2024a; da Costa et al. 2025). This strategy also helps lower the risk of antimicrobial resistance, which is often promoted by the prophylactic treatments prescribed in these cases (Flores-Mireles et al. 2019).

Indeed, drugs acting on the central nervous system, such as selective serotonin reuptake inhibitors and tricyclic antidepressants, have already demonstrated in vitro activity against *Candida* biofilms (Begec et al. 2013; Sá et al. 2019; de Andrade Neto et al. 2020; Holanda et al. 2021).

Ketamine (KET), a phencyclidine derivative widely used as a dissociative anesthetic, possesses well-documented analgesic, anti-inflammatory, and antidepressant properties (Cavalcanti et al. 2020; Kurdi et al. 2014; Zanos et al. 2018). Previous studies have demonstrated the antifungal activity of KET [24, 31], and notably its synergistic potential when combined with traditional azole antifungals such as fluconazole (FLC) (de Andrade Neto et al. 2020).

Given the growing threat posed by *Candida* biofilms resistant to conventional treatments, we investigated the ability of KET, both alone and in combination with the azoles FLC and itraconazole (ITR), to inhibit the colonization of *C. albicans* on impregnated catheter surfaces. Furthermore, we specifically evaluated the effects on biofilm formation and viability on plates, as well as the structural alterations induced by the treatments using scanning electron microscopy (SEM).

## Methodology

### Drugs

The drugs used in this study were KET, ITR, and FLC (Sigma-Aldrich, St. Louis, MO, USA). The minimum inhibitory concentration (MIC) values used in this study were previously determined in our earlier work using the broth microdilution method, as described by de Andrade-Neto et al. (2020), where the MIC values 3.73 mM for KET, 0.000354 mM for ITR, and ranged from 0.1208 to 0.1926 mM for FLC. Based on these results, MIC and 2×MIC concentrations were applied in the TSA assays analyzed here.

### Microorganisms

We selected three strains of *Candida* spp. for this study, namely one *C. albicans*, one *C. tropicalis*, and one *C. parapsilosis*. All strains are part of the fungal collection of the Laboratory for Bioprospecting of Antimicrobial Molecules (LABIMAN) at Federal University of Ceará (UFC), Fortaleza, Brazil, and were preserved at -20 °C in Sabouraud broth (HiMedia, Mumbai, India) supplemented with glycerol (Dinâmica, São Paulo, Brazil). For reactivation, the strains were inoculated into Sabouraud broth (HiMedia) and incubated at 35 °C for 24 h. Subsequently, they were plated on Sabouraud dextrose agar (HiMedia) and incubated under the same conditions (35 °C, 24 h) to verify the viability and purity of the cultures (Neto et al. 2014; Sá et al. 2020).

### Biofilm formation inhibition assay

Biofilm formation was carried out following the method described by Pierce et al. (2008), with modifications. Initially, yeast cultures incubated for 24 h at 35 °C were centrifuged at 2500 rpm for 5 min. The resulting cell pellet was washed three times with 3 mL of phosphate-buffered saline (PBS). After the final washing and removal of the supernatant, the pellet was resuspended in 500 µL of PBS. From this suspension, a standardized inoculum was prepared at 0.5 on the McFarland scale in RPMI 1640 broth medium (pH 7.0) buffered with 0.165 M MOPS (Sigma-Aldrich). In each microplate well, 100 µL of this inoculum was added to 100 µL of the drug solutions (KET, FLC, ITR) or their combinations (KET + FLC; KET + ITR), at MIC and 2× MIC concentrations. After 24 h of incubation at 35 °C, the culture medium was removed, and the adhered biofilm was washed with PBS to eliminate non-adherent cells. To quantify viable biomass, 3-(4,5- dimethylthiazol-2-yl)-2,5-diphenyltetrazolium bromide (MTT) solution was added to each well, followed by incubation for 24 h at 35 °C. Subsequently, the MTT solution was discarded, and 150 µL of DMSO was added to solubilize the formazan crystals that had formed. Finally, 100 µL of the resulting solution was transferred to each well of a new microplate, and absorbance was measured using a Smartreader^tm^ 96 microplate reader (Accuris Instruments, NJ, USA) at a wavelength of 570 nm.

### Preparation of catheter fragments

For the analysis involving impregnated catheters, a representative strain (*C. albicans*) was selected due to its epidemiological relevance. The method used to evaluate the effect of catheter impregnation on the prevention of fungal biofilm formation was adapted from Silva et al. (2024b). Sterile 0.5 cm segments were cut from a peripheral venous catheter (Jelco BD 18G Angiocath intravenous catheter). For impregnation, the segments were individually submerged in 1 mL of chloroform containing the following substances: KET, FLC, ITR, and the combinations KET + FLC and KET + ITR. All substances and combinations were used at concentrations equivalent to twice the minimum inhibitory concentration (2× MIC), with MIC values based on the results reported by de Andrade-Neto et al. (2020). A control group was prepared by submerging segments in 1 mL of pure chloroform only. After 24 h of submersion, the segments were aseptically removed, and the chloroform was allowed to evaporate completely in a sterile laminar flow hood at room temperature.

### Catheter fragment impregnation assay

The impregnated catheter segments (treated and solvent control) were individually transferred to wells of sterile 24-well polystyrene plates. Each well received 200 µL of fungal inoculum, and the plates were incubated at 37 °C for 48 h. After the incubation period, the supernatant was carefully removed, and the catheter segments were washed twice with sterile saline solution (0.85% NaCl) to eliminate non-adherent fungal cells.

To quantify the viable cells adhered to the catheter samples, each washed segment was transferred to a sterile tube containing 1 mL of saline solution (0.85% NaCl). The adhered cells were dislodged by vigorous vortexing for 1.5 min. The resulting cell suspension was then serially diluted in saline solution to a final dilution factor of 1:1000. Aliquots of 10 µL from the 1:1000 dilution were surface-plated onto potato dextrose agar (PDA; Kasvi, Paraná, Brazil) supplemented with chloramphenicol (Sigma-Aldrich, St. Louis, MO, USA) to inhibit bacterial growth. The plates were incubated at 37 °C for 24 h. Finally, colonies were counted, and the results were calculated and expressed as colony forming units per milliliter (CFU/mL) (Iwata et al. 2021; Silva et al. 2024b).

### Scanning electron microscopy (SEM)

*C. albicans* biofilms were fixed with 2.5% glutaraldehyde in 0.15 M sodium cacodylate buffer containing 0.1% Alcian blue, incubated overnight at 4 °C, dehydrated through a graded ethanol series, dried with hexamethyldisilazane (Policiências Europa, Hirschberg an der Bergstraße, Germany), coated with a 10 nm layer of gold (Emitech Q150T, Quorum Technologies Ltd, Laughton, United Kingdom), and examined using an FEI Inspect S50 scanning electron microscope (Brno, Czech Republic) operating in high vacuum mode at 15 Kv (Begec et al. 2013).

### Statistical analysis

All assays were performed in triplicate across three independent experiments. To compare the mean absorbance values from the biofilm growth assay, one-way analysis of variance (ANOVA) was used, followed by the Tukey post hoc test (*p* < 0.05). All analyses were conducted using GraphPad Prism (version 7.03 for Windows, GraphPad Software, La Jolla, CA, USA).

## Results

### Inhibition of biofilm formation by KET alone and in combination with azoles against *Candida* spp., assessed by the MTT assay

**Fig. 1:**
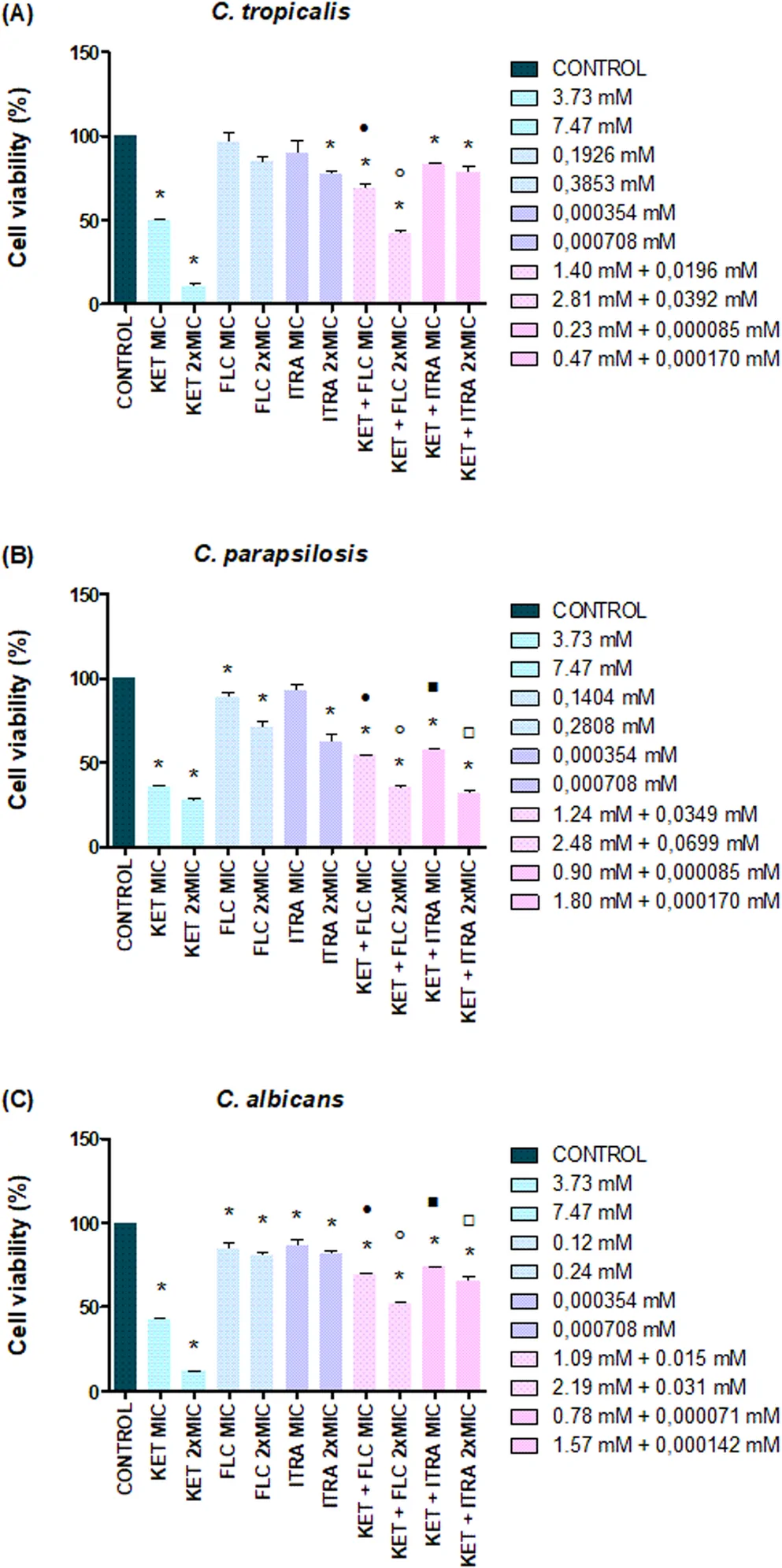
Activity of KET, FLC, ITR, and their pairwise combinations on biofilms formed by *Candida* species, evaluated by the MTT reduction assay. *P < 0.05: Results compared to the growth control. •*P* < 0.05: Results compared to FLC at MIC. °*P* < 0.05: Results compared to FLC at 2× MIC. ■*P* < 0.05: Results compared to ITR at MIC. □ *P* < 0.05: Results compared to ITR at 2× MIC. Statistical significance was determined using one-way ANOVA followed by Tukey’s post hoc test. (**A**) Cell viability of *C. tropicalis* biofilm. (**B**) Cell viability of *C. parapsilosis* biofilm. (**C**) Cell viability of *C. albicans* biofilm. For *C. tropicalis*, the MIC concentrations of the drugs were: KET (3.73 mM) FLC (0.1926 mM); ITR (0.000354 mM), KET + FLC (KET 1.40 mM + FLC 0.0196 mM), KET + ITR (KET 0.23 mM + ITR 0.000085 mM), and at 2x MIC: KET (7.47 mM), FLC (0.3853 mM); ITR (0.000708 mM), KET + FLC (KET 2.81 mM + FLC 0.0392 mM), KET + ITR (KET 0.47 mM + ITR 0.000170 mM). For *C. parapsilosis*, the MIC concentrations of the treatments were: KET (3.73 mM), FLC (0.1404 mM), ITR (0. 000354 mM) KET + FLC (KET 1.24 mM + FLC 0.0349 mM), KET + ITR (KET 0.90 mM + ITR 0.000085 mM), and at 2x MIC: KET (7.47 mM), FLC (0.2808 mM), ITR (0.000708 mM), KET + FLC (KET 2.48 mM + FLC 0.0699 mM), KET + ITR (KET 1.80 mM + ITR 0.000170 mM). Finally, for *C. albicans*, the MIC concentrations were: KET (3.73 mM), FLC (0.12 mM), ITR (0.000354 mM), KET + FLC (KET 1.09 mM + FLC 0.015 mM), KET + ITR (KET 0.78 mM + ITR 0.00071 mM), and it 2x MIC: KET (7.47 mM), FLC (0.24 mM), ITR (0.000708 mM), KET + FLC (KET 2.19 mM + FLC 0.031 mM), KET + ITR (KET 1.57 mM + ITR 0.000142 mM)

Figure 1A shows biofilm formation by *C. tropicalis*, where the combinations KET + FLC and KET + ITR resulted in 68.6% ± 4.07% and 82.6% ± 2.50% viability, respectively. Upon increasing the concentrations to 2× MIC levels, significant attenuation of viability was observed (*p* < 0.05): KET reduced viability to 15.53% ± 1.91%; KET + FLC to 42.5% ± 1.67%; and KET + ITR to 82.67% ± 2.5%. The combinations were significantly (*p* < 0.05) more effective than the azoles alone (FLC and ITR).

Regarding *C. parapsilosis*, KET + FLC and KET + ITR showed 54.47% ± 0.72 and 57.75% ± 1.57% viability, respectively. Again, the 2× MIC concentrations were more potent (*p* < 0.05): KET resulted in 27.54% ± 2.10% viability; KET + FLC in 35.23% ± 1.12%; and KET + ITR in 31.88% ± 2.26%. All the combinations outperformed the efficacy of the azoles alone (*p* < 0.05) (Fig. 1B).

Against biofilms formed by *C. albicans*, KET + FLC and KET + ITR exhibited 69.33% ± 0.87% and 73.86% ± 0.10% viability, respectively. The effects were significantly greater at 2× MIC concentrations (*p* < 0.05): KET reduced viability to 11.78% ± 0.85%; KET + FLC to 52.11% ± 2.10%; and KET + ITR to 65.68% ± 3.75%. The combinations with KET were more effective than FLC or ITR alone (*p* < 0.05) (Fig. 1C).

### Evaluation of biofilm formation inhibition by KET in impregnated catheters

**Fig. 2:**
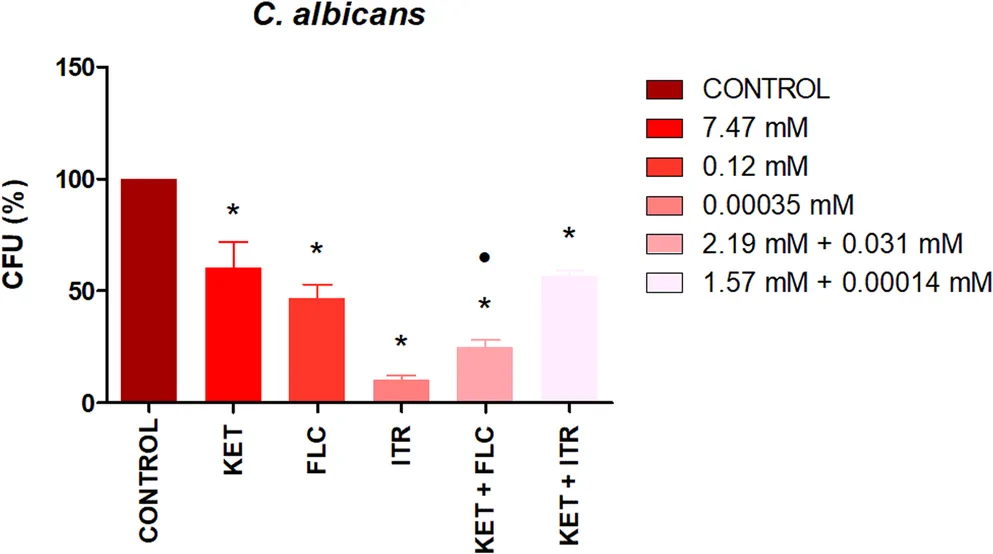
Graphical representation of the percentage of colony forming units (CFU%) of *Candida albicans* that adhered to catheter segments after treatment. The catheter samples were impregnated with KET (7.47 mM), FLC (0.12 mM), ITR (0.00035 mM), and the combinations KET + FLC (KET 2.19 mM + FLC 0.031 mM) and KET + ITR (KET 1.57 mM + ITR 0.00014 mM) at 2× MIC concentrations. Bars represent the mean ± standard error of the mean (SEM). *P < 0.05: Results were compared to the growth control. •*P* < 0.05: Results were compared to FLC at 2× MIC

The ability of catheter segment impregnation with KET, FLC, ITR, and their combinations (KET + FLC, KET + ITR), at concentrations corresponding to 2× MIC, to inhibit *C. albicans* adherence was assessed by quantifying CFU. One-way analysis of variance (ANOVA) indicated a highly significant difference among treatment groups (*p* < 0.0001). All treatments promoted a statistically significant reduction in adherence compared to the control group (*p* < 0.05). Among the individual treatments, KET resulted in a mean reduction of 39.6%, FLC in 53.7%, and ITR in 89.8%. Regarding the combined treatments, KET + FLC promoted a reduction of approximately 35.4% and 21.3% in CFU/mL of *Candida* albicans compared to the isolated treatments (Fig. 2).

### Evaluation of biofilm formation inhibition by scanning electron microscopy (SEM)

**Fig. 3:**
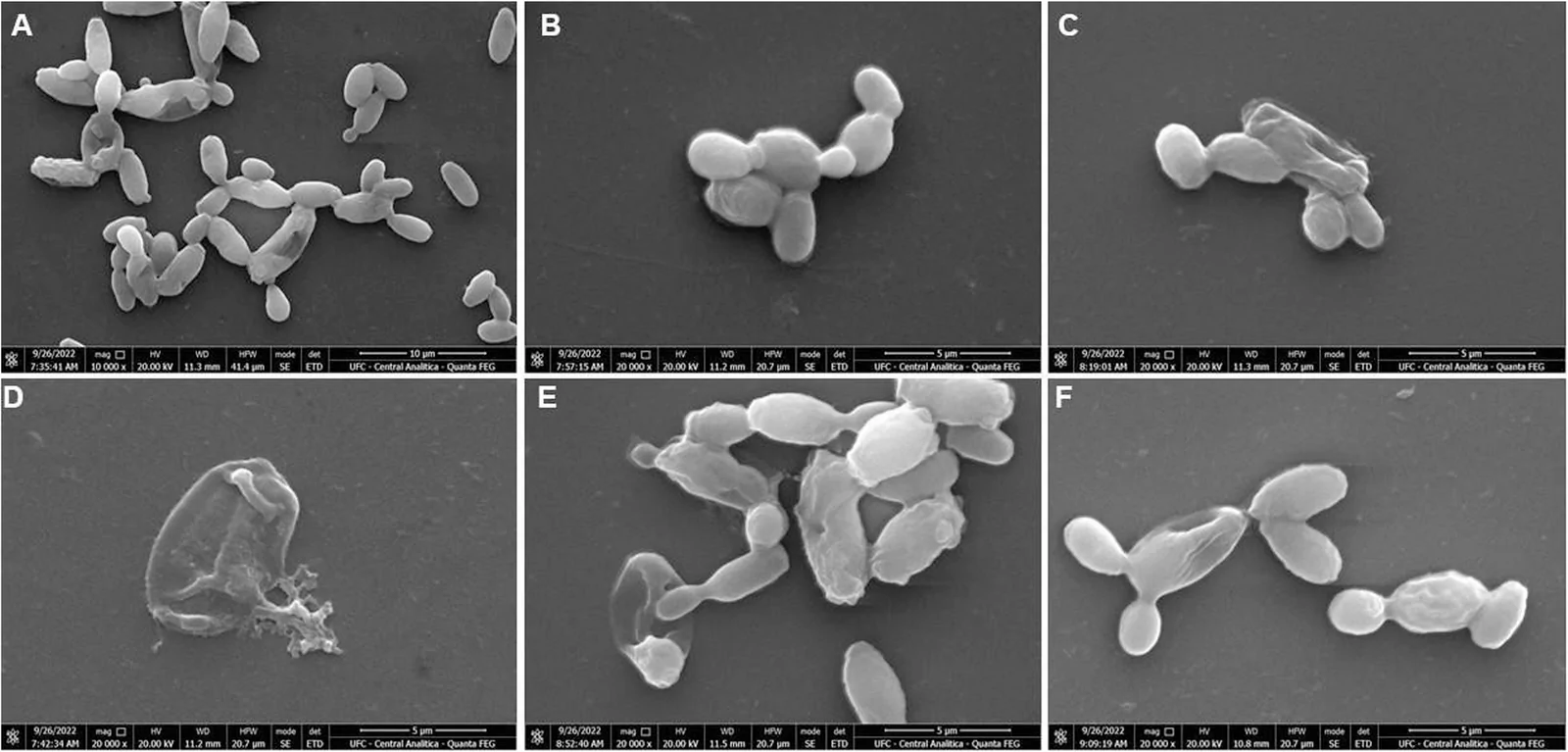
Scanning electron micrographs (SEM) illustrating the effects of treatments on *Candida albicans* biofilm formation. Cells were exposed to treatments at MIC during biofilm development: (**A**) Control (untreated), showing aggregated cells with normal morphology; (**B**) Fluconazole (FLC); (**C**) Itraconazole (ITR); (**D**) Ketamine (KET), exhibiting severe cellular damage and collapse; (**E**) KET + FLC; (**F**) KET + ITR. Scale bars: (**A**) 10 µm (10,000x); (**B**-**F**) 5 µm (20,000x)

SEM was employed to assess the cellular morphology and biofilm architecture of *C. albicans* following in vitro treatments. The control group exhibited dense microcolonies of yeast-like cells with well-preserved morphology (Fig. 3A). Treatments with FLC (Fig. 3B) and ITR (Fig. 3C) resulted in reduced cell density. Treatment with KET (Fig. 3D) induced extensive cellular damage, characterized by collapsed and irregular cells. The combinations KET + FLC (Fig. 3E) and KET + ITR (Fig. 3F) resulted in cells with better-preserved morphology compared to KET alone.

### Adherence to impregnated catheters

**Fig. 4:**
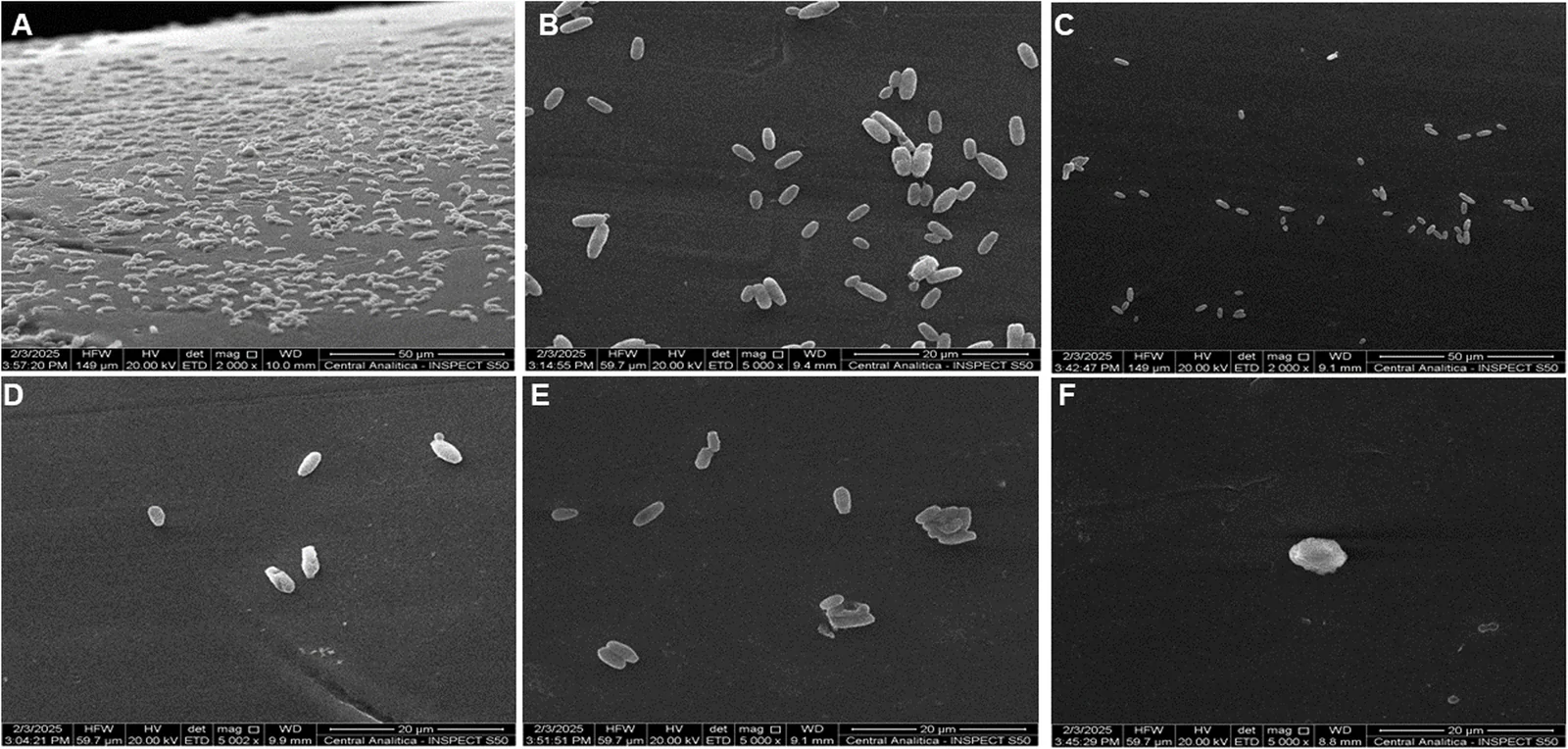
Scanning electron micrographs (SEM) of *Candida albicans* adherence to catheter segments after 48 hours of incubation. (**A**) Control (non-impregnated) catheter sample, showing dense surface colonization. (B-F) Catheters pre-impregnated with treatments at 2× MIC: (**B**) Fluconazole (FLC); (**C**) Itraconazole (ITR); (**D**) Ketamine (KET); (**E**) KET + FLC; (**F**) KET + ITR. A significant reduction in cellular adherence was observed on all treated surfaces, particularly in (**C**), (**D**) and (**F**). Scale bars: (**A**) 50 µm (2.000x); (**B**-**F**) 20 µm (5.000x)

**Fig. 5:**
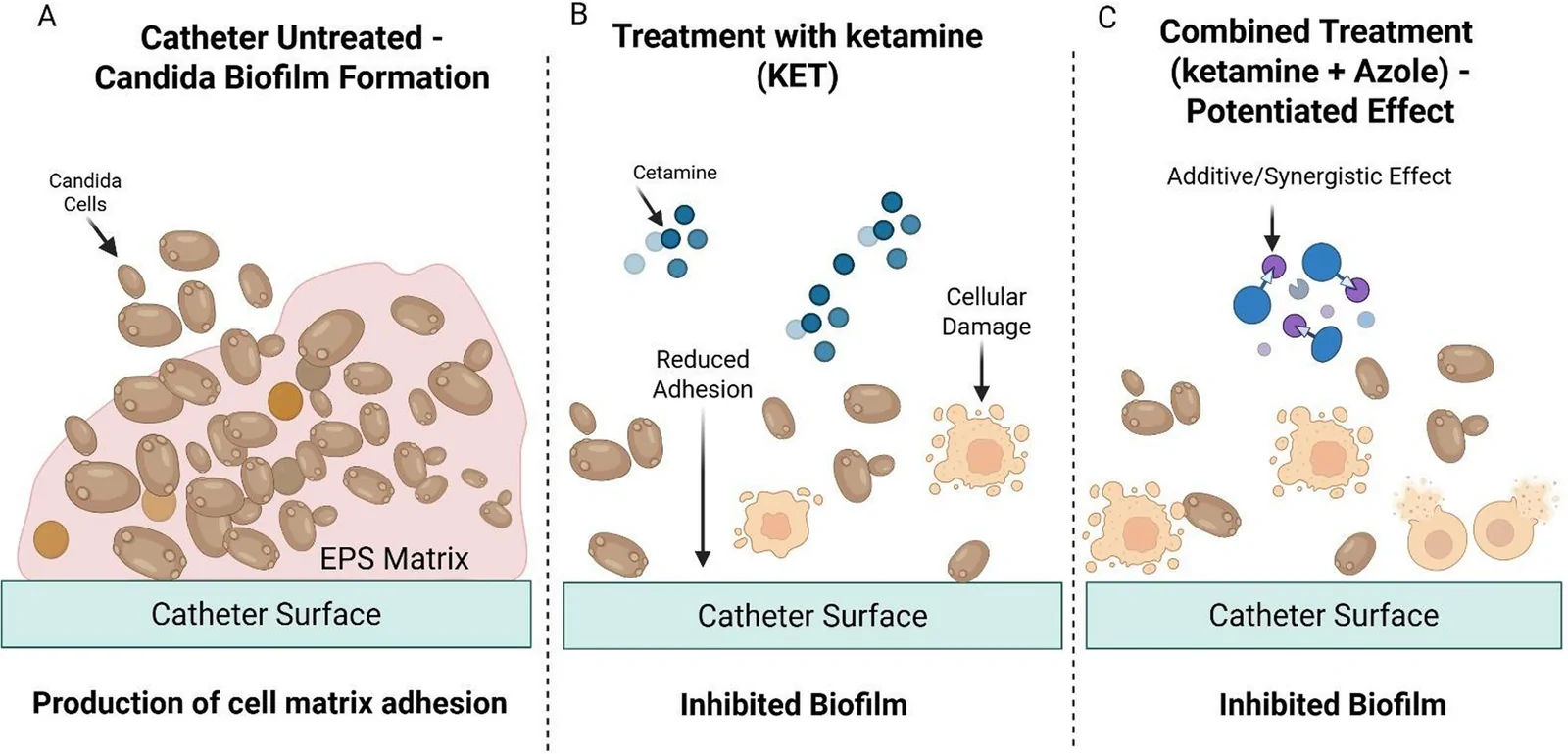
Conceptual representation of the antibiofilm activity of ketamine against *Candida albicans* on catheters, based on the viability assays, adherence studies, and electron microscopy analyses presented (SEM) in this study. (**A**) Untreated mature biofilm. (**B**) Ketamine (KET) reduced adherence, inhibited biofilm formation, and caused cellular damage. (**C**) KET + Azole (Fluconazole/Itraconazole) enhanced inhibition, resulting in minimal colonization (additive/synergistic effect). Created with BioRender.com

SEM was used to evaluate cellular adherence to catheter segments impregnated at 2× MIC. Control catheters exhibited dense and confluent cellular colonization (Fig. 4A). All impregnation treatments drastically reduced cellular adherence. The most pronounced inhibition was observed in catheters impregnated with ITR (Fig. 4C), KET (Fig. 4D), and KET + ITR (Fig. 4F), which displayed surfaces with very sparse adhered cells. FLC (Fig. 4B) and KET + FLC (Fig. 4E) also reduced adherence, but with a greater number of remaining cells, either isolated or in small clusters, compared to the other treatments. The adhered cells on the treated surfaces maintained normal morphology. Taken together, the data presented demonstrate the ability of KET to act against *C. albicans* biofilms, both during their formation and their adherence to clinically relevant surfaces such as catheters—an effect that may be enhanced by azole antifungals. Figure 5 provides a schematic representation of these findings, consolidating the observations. The diagram illustrates robust biofilm formation in the control group (Fig. 5A), in contrast with the significant reduction in colonization and cellular damage induced by KET alone (Fig. 5B), as observed in the viability assays (Fig. 1) and SEM analysis (Fig. 3D). Additionally, it depicts the even more pronounced inhibition of adherence and biofilm formation promoted by the KET + FLC and KET + ITR combinations (Fig. 5C), consistent with the CFU quantification results (Fig. 2) and SEM adherence observation (Fig. 4).

## Discussion

Biofilm formation by *Candida* species, particularly on medical devices, represents a significant clinical challenge, as it confers drug tolerance and protection against host defenses (Cavalheiro and Teixeira 2018; Ponde et al. 2021). In this context, coating such devices with antimicrobial molecules has emerged as a promising strategy to reduce microbial colonization and hinder biofilm establishment (Reginatto et al. 2020). This approach is especially relevant in the context of catheter-related bloodstream infections, in which *Candida* biofilm formation plays a central role in pathogenesis (Thomas-Rüddel et al. 2022; Ozalp and Ozturk et al. 2025).

Due to the need for effective strategies to control these infections, our results not only corroborate the antimicrobial activity of KET, previously observed against *Staphylococcus aureus* (Coutinho et al. 2021) and *Stachybotrys chartarum* (Torres et al. 2018), but also significantly extend this finding by demonstrating its efficacy against major human pathogens such as *C. albicans* and *C. tropicalis*, particularly in biofilms, a widely recognized therapeutic challenge.

Promisingly, our findings also suggest a synergistic effect between KET and the azole antifungals ITR and FLC in inhibiting the formation and persistence of *Candida* biofilms. This synergistic interaction opens relevant clinical perspectives, as the potential use of lower doses of both drugs could reduce their individual toxicity profiles, thereby improving treatment tolerance by vulnerable patients (Li et al. 2024).

Additionally, this strategic combination may represent an effective approach to overcome the increasing resistance to azoles by restoring susceptibility in previously resistant strains. The combined action may also lead to a more efficient disruption of the complex extracellular matrix of the biofilm, which is a key factor in the persistence of fungal infections, thereby facilitating infection eradication (Chen et al. 2014; Gómez-Gaviria et al. 2024).

It is relevant to note that a synergistic effect between KET and azoles against *Candida* biofilms has already been reported (de Andrade Neto et al. 2020), corroborating our findings and proposing the induction of reactive oxygen species (ROS) as a possible underlying mechanism. Although the literature on the direct interaction between anesthetics and antifungals is still limited, the understanding of individual mechanisms of action suggests that KET’s ability to induce oxidative stress may compromise the integrity of the fungal cell membrane, making it more vulnerable to the inhibition of ergosterol synthesis by azoles (de Andrade Neto et al. 2020). Future studies focused on elucidating the precise molecular mechanisms that govern this synergy are therefore essential to fully explore the therapeutic potential of this innovative combination.

ROS are considered essential regulators in the human aging process and have also been reported as important elements involved in fungal apoptosis, as observed by Hwang et al. (2012). ROS function as signal transducers and accumulate within the cell in the form of hydrogen peroxide, superoxide radicals, and hydroxyl radicals. These forms are closely associated with cellular disintegration and also help explain the disruption process of *Candida* spp. biofilms (Hwang et al. 2012). Although the in vitro data from Cavalcanti et al. (2020) suggest low genotoxicity, the authors themselves noted that in vivo studies were scarce. Further investigations are necessary to assess the effects of chronic exposure and/or to explore outcomes in complex animal models.

Antimicrobial activity does not appear to be exclusive to KET within the class of anesthetics. Studies involving etomidate (Sá et al. 2019) and combinations of KET + propofol (Begec et al. 2013) have also reported effects against *Candida* and/or bacteria. Notably, Sá et al. (2020) demonstrated that etomidate inhibits biofilms of *Candida* spp. resistant to FLC and interacts in silico with the adhesin *ALS3*, in addition to reducing the availability of mannoproteins. This convergence of findings involving different anesthetics suggests they may modulate fungal physiology through mechanisms that warrant further investigation.

SEM analysis provided morphological support for the viability data, revealing significant structural alterations in biofilms treated with KET. We observed evident cellular damage, such as collapse and surface irregularities, in *Candida* cells exposed to KET (Fig. 3D), which are indicative of compromised cellular integrity. These morphological changes are consistent with the hypothesis that KET induces cellular stress, potentially through the generation of ROS, a mechanism previously proposed for this compound (de Andrade Neto et al. 2020). The accumulation of ROS is a known mediator of macromolecular damage and an inducer of apoptosis in fungi, which may contribute to the destabilization of the biofilm structure (Gonçalves et al. 2017). Interestingly, this proposed mechanism for KET differs from observations regarding another anesthetic with antibiofilm activity, midazolam. Although Holanda et al. (2021) detected apoptotic markers such as mitochondrial depolarization and phosphatidylserine externalization in *Candida* treated with midazolam, they did not observe a significant increase in ROS production or DNA damage. This apparent divergence of mechanisms suggests that, although different classes of anesthetics may convergently induce cell death pathways in fungi within biofilms, the initial molecular triggers, specifically the role of oxidative stress, may vary, thus warranting further investigation of each compound.

A significant aspect of our study lies in demonstrating that the impregnation of catheter segments with KET, either alone or in combination (KET + ITR and KET + FLC), markedly inhibited the initial adherence of *C. albicans* (Fig. 4D, E and F). Since the colonization of medical devices is a critical step in the pathogenesis of many biofilm-associated infections, preventing this initial stage is a key prophylactic strategy (Francolini and Donelli 2010; Zhang et al. 2020; Li et al. 2023). Our findings add to those reported by Silva et al.(2024b), who described the efficacy of etomidate-impregnated catheters in preventing polymicrobial biofilms (*C. albicans*–MRSA), reinforcing the potential applicability of anesthetics in this context.

Despite these promising findings, some limitations should be acknowledged. Our investigations were conducted exclusively in vitro, which may not fully replicate the complexity of the in vivo environment. Although we observed significant morphological damage, as well as reduced viability and adhesion, the precise molecular mechanisms underlying KET’s activity and the nature of its additive or synergistic interactions with azoles remain to be elucidated. Future studies involving gene expression analyses and in vivo models will be essential to clarify these mechanisms and validate the translational potential of our findings. Nevertheless, the robustness of this study is supported by the evaluation of multiple *Candida* species, the investigation of combination therapies, and the use of a clinically relevant abiotic surface model (catheter), which enhances the applicability of the results.

In summary, our findings position KET as a promising candidate for drug repurposing in the management of *Candida* biofilms. Its intrinsic antifungal activity, its ability to potentiate conventional antifungal agents, and its inhibitory effect on catheter colonization highlight its potential as a novel adjunctive therapeutic strategy. In addition, these results support the exploration of KET based approaches, such as catheter coating or lock therapies, aimed at preventing device-associated infections. In this context, the present study contributes to advancing the understanding of antibiofilm strategies and underscores the relevance of drug repurposing as an alternative approach to tackling device-associated infections.

## Data Availability

No datasets were generated or analysed during the current study.
